# Variations in dynamic tumor-associated antigen-specific T cell responses correlate with HCC recurrence after thermal ablation

**DOI:** 10.3389/fimmu.2022.982578

**Published:** 2022-12-22

**Authors:** Chaoran Zang, Yan Zhao, Guihai Liu, Kang Li, Ling Qin, Yuewei Zhang, Jianping Sun, Qi Wang, Liang Ma, Peng Zhao, Yu Sun, Dandan Guo, Chunwang Yuan, Tao Dong, Yonghong Zhang

**Affiliations:** ^1^ Interventional Therapy Center of Liver Disease, Beijing YouAn Hospital, Capital Medical University, Beijing, China; ^2^ Pancreatic Center Department, Beijing Tsinghua Changgung Hospital, School of Clinical Medicine, Tsinghua University, Beijing, China; ^3^ Biomedical Information Center, Beijing YouAn Hospital, Capital Medical University, Beijing, China; ^4^ Clinical Laboratory Center, Beijing YouAn Hospital, Capital Medical University, Beijing, China; ^5^ Medical Research Council (MRC) Human Immunology Unit, Medical Research Council (MRC) Weatherall Institute of Molecular Medicine, Oxford University, Oxford, United Kingdom; ^6^ Chinese Academy of Medical Sciences (CAMS) Oxford Institute, Nuffield Department of Medicine, Oxford University, Oxford, United Kingdom

**Keywords:** hepatocellular carcinoma, ablation, tumor-associated antigen, T-cell immune response, recurrence

## Abstract

**Background:**

Ablative therapy is a recommended treatment for hepatocellular carcinoma (HCC) not only for its effective eradication of tumors, but also for its induction of host immunity. However, the high 5-year recurrence rate after ablation underlines the poor understanding of the antitumor immunity response. Here, we investigated the effects of thermal ablation on antitumor immunity.

**Methods:**

We analyzed the dynamics of tumor-associated antigen (TAA)-specific immune responses and changes in peripheral blood mononuclear cell phenotype in patients with HCC before and after tumor ablation. We used the IFN-γ ELISPOT assay and immunophenotyping by flow cytometry to evaluate the effects of ablation on host immunity. The correlation between the T cell response and disease outcome was explored to uncover the efficacy of the immune response in inhibiting HCC recurrence.

**Results:**

Different TAA-specific T cell responses were identified among patients before and after ablation. One week after ablation, there was an improved immune state, with a switch from the dominance of an AFP-specific T cell response to that of a SMNMS-specific T cell response, which was correlated with better survival. Furthermore, an improvement in immune status was accompanied by a lower level of PD1+ and Tim3+ T cells in CD8+ T cells. Although this functional state was not durable, there was a higher degree of AFP-specific T cell responses at 4-weeks post-ablation. Furthermore, T cells presented a more exhausted phenotype at 4-weeks post-ablation than at the 1-week timepoint.

**Conclusions:**

Ablation elicits a transient antitumor immune response in patients with HCC by changing the profile of the T cell response and the expression of immune checkpoint molecules, which correlated with longer recurrence-free survival of patients with HCC.

## Introduction

As the sixth most common neoplasm and the third leading cause of cancer death, hepatocellular carcinoma (HCC) is a disease that seriously threatens human health (1). Currently, ablative therapy has been recommended as the first-line treatment for HCC of Barcelona Clinic Liver Cancer (BCLC) stage 0/A according to the EASL Clinical Guidelines (2). At the same time, due to the obvious advantages of ablative therapy, its application is also gradually broadening (3). More interestingly, the abscopal effect after ablation indicates the induction of anti-tumor immunity by the destruction of tumours (4). Previous studies have also suggested that there is an increase in systemic antitumor immunity following ablation (5–9), including both in terms of innate immunity (10, 11) and adaptive immunity (6, 7, 12). However, in some patients with HCC, early recurrence after ablation leads to a dismal 5-year survival rate (1), which also indicates that understanding of this immune induction is insufficient.

The T-cell response plays an important role in the control of tumor progression by preventing or controlling tumor growth (13, 14). Flecken et al. showed that TAA-specific CD8+ T cell responses were associated with prolonged progression-free survival in patients with HCC (15). Our previous study found that broader and stronger SALL4, MAGE-A1, NY-ESO-1, MAGE-A3, and SSX2 (SMNMS)-specific T cell responses correlated with early-stage HCC, while a single T cell response, especially that of α-fetoprotein (AFP)-specific T cells, emerged mainly in the advanced stages (14). Furthermore, patients with a higher SMNMS-specific T cell response achieved a better 1-year recurrence-free survival (RFS).

Although encouraging data have been reported (3, 13), tumor recurrence in patients with HCC who have undergone complete ablation is still inevitable. To what extent can ablative therapy alter the HCC-specific T cell response? Furthermore, how long can the ablation-induced HCC-specific T cell response last? To address these issues, we studied the dynamics of tumor-specific T cells induced by ablative therapy during a specific follow-up period post-ablation using SALL4, MAGE-A1, MAGE-A3, NY-ESO-1, SSX2 and AFP to stimulate the antitumor T cell response. We analyzed the correlation between tumor-associated antigen (TAA)-specific T cell response and the recurrence of HCC after ablation.

## Methods

### Subjects of study

**Figure 1:**
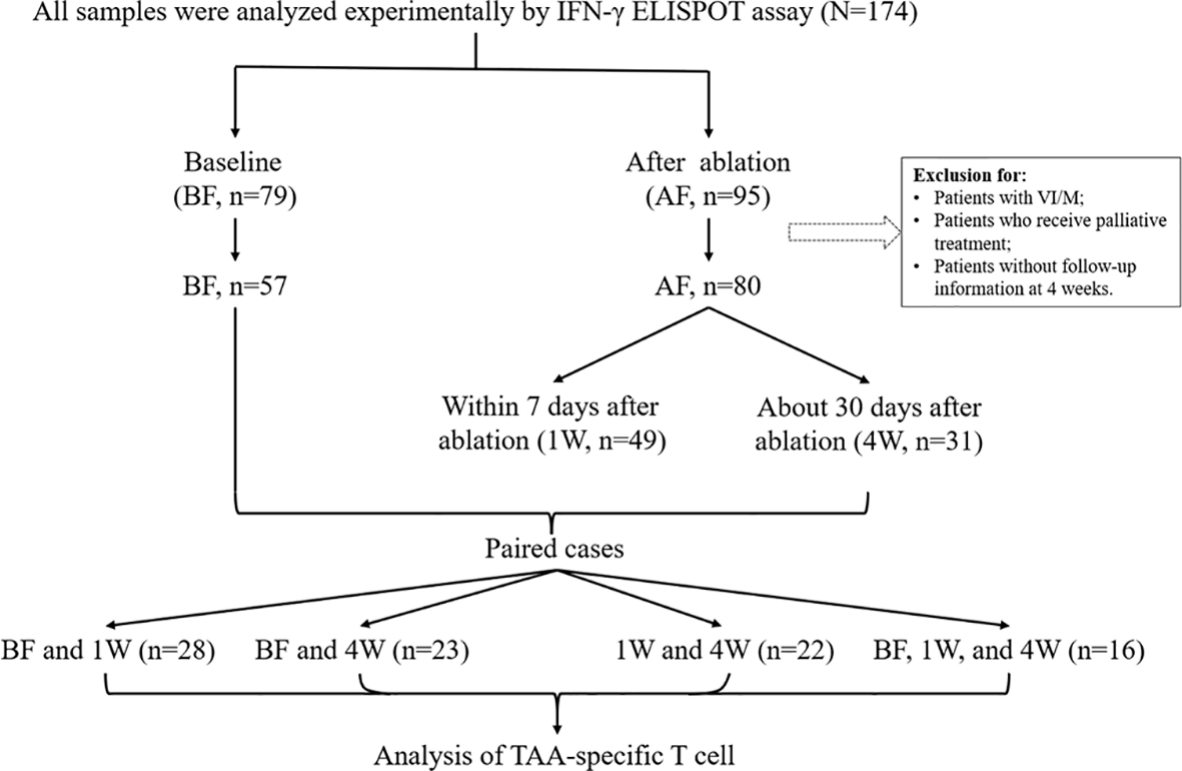
Patient cohort and study design. 174 HCC PBMCs were initially included in this study. Among them, 79 samples were before treatment (BF), and 95 samples were that had received ablation treatment (AF). In consideration of the disease condition and the effect of treatment, samples from patients with vascular invasion/metastasis and without curative therapy were excluded. Thus, there were 57 samples at BF and 80 samples at AF. In the AF group, there were 49 samples were enrolled at 1-week (1W) and 39 samples at 4-week (4W). Paired cases were analyzed in 28 samples at BF and 1W, in 23 samples at BF and 4W, in 22 samples at 1W and 4W, two timepoints, and 16 samples at BF, 1W and 4W, three timepoints. Clinical data were collected from the database.

A total of 174 HCC samples were initially included from 2017/7 to 2021/7 in Beijing YouAn Hospital in this study. The diagnosis of HCC was histological confirmed or based on typical hypervascular tumour staining on angiography in addition to typical findings, which showed hyperattenuated areas in the early phase and hypoattenuation in the late phase on dynamic computed tomography (CT) or magnetic resonance imaging (MRI) (2). The following inclusion criteria were used: 1) liver biopsy or film degree exam diagnosed as HCC; 2) age from 18 to 75 years; 3) liver cirrhosis classified as Child−Pugh class A or B; 4) no other malignancies that may affect the prognosis. The exclusion criteria were as follows: 1) subjects who have received immune-related treatment; 2) with coexistent hematological disorders, serious or active infection before treatment; 3) combinations with other types of cancer or autoimmune disease; 4) secondary liver cancer; 5) serious treatment-related complications developed; 6) patients who developed tumor thrombus or metastasis; 7) tumours were not necrotic completely when assessed 4 weeks post-ablation. Therefore, during the course of our project, 174 samples were collected, including 79 baseline samples and 95 postoperative samples. After exclusion according to the standard, there were 57 preoperative samples and 80 postoperative samples. Next, we divided all the samples into three time points: 57 samples at before treatment (BF), 49 samples at 1 week (1W) and 31 samples at 4 weeks (4W) after treatment. In the following analysis, we compared changes in the T-cell immune response before and after treatment in each patient, for example, BF:1W, BF:4W, 1W:4W, and BF:1W:4W matched data, and there were 28, 23, 22, and 16 patients, respectively. The study design is outlined in **Figure 1**.

All subjects had undergone abdominal CT or abdominal MRI before and after thermal ablation (thereafter referred to as ablation). All patients gave written informed consent to participate in the study in accordance with the Helsinki declaration, and this study was approved by The Ethics Committee of Beijing YouAn Hospital, Capital Medical University.

### Interventional treatments

All candidates enrolled in the study were performed combination therapy with transcatheter arterial chemoembolization (TACE) and thermal ablation (radiofrequency ablation or microwave ablation according to the assessment of tumour conditions). In the TACE procedure, the microcatheter was selectively/super-selectively placed in the tumour-feeding artery. A mixture of doxorubicin (Pfizer Inc., NY, USA) and lipiodol (Guerbet, Villepinte, France) was injected, and Gelfoam was used for embolization. Occlusions of the feeding artery and disappearance of the vessel stain were identified as the endpoint of embolization. Local thermal ablation was performed within 1 week after TACE. With the guidance of CT or MRI, the ablative position and modality were determined. Multiple sites, overlapping ablation, and repeated ablation were considered according to the tumour number and size to achieve the best clinical effect. For patients undergoing curative treatment, a safety margin of 0.5–1.0 cm of the adjacent non-neoplastic tissue was ablated to ensure complete coverage. The aforementioned treatments were performed by an interventional radiologist with >5 years of experience.

### Samples collection

A total of 10 ml whole−blood samples were collected before (BF) or after (AF) treatment. Peripheral blood mononuclear cells (PBMCs) were isolated by Ficoll density gradient within 6 hours after peripheral blood collection, then were resuspended cell cryopreservation fluid containing 90% fetal calf serum and 10% dimethyl sulfoxide and stored in liquid nitrogen until use.

### Synthetic peptides for T-cell analysis

A total of 334 overlapping peptides (18-mers overlapping by 10 amino acids) spanning the complete amino acid sequence of SALL4, MAGE-A1, MAGE-A3, NY-ESO-1, SSX2 and AFP were utilized. Their purities were determined to be >90% by analytical high-performance liquid chromatography. Peptides were dissolved in dimethylsulfoxide (Sigma, Haverhill, Suffolk, UK) and diluted with RPMI 1640 before being combined into nine pools with 23-45 peptides per pool (**Table 1**).

**Table 1:** The sequences of the overlapping peptides for each of the six antigens and their position within the protein sequence are shown.

List of overlapping peptides	
peptide	sequence
AFP-1	MKWVESIFLIFLLNFTES
AFP-2	LIFLLNFTESRTLHRNEY
AFP-3	ESRTLHRNEYGIASILDS
AFP-4	EYGIASILDSYQCTAEIS
AFP-5	DSYQCTAEISLADLATIF
AFP-6	ISLADLATIFFAQFVQEA
AFP-7	IFFAQFVQEATYKEVSKM
AFP-8	EATYKEVSKMVKDALTAI
AFP-9	KMVKDALTAIEKPTGDEQ
AFP-10	AIEKPTGDEQSSGCLENQ
AFP-11	EQSSGCLENQLPAFLEEL
AFP-12	NQLPAFLEELCHEKEILE
AFP-13	ELCHEKEILEKYGHSDCC
AFP-14	LEKYGHSDCCSQSEEGRH
AFP-15	CCSQSEEGRHNCFLAHKK
AFP-16	RHNCFLAHKKPTPASIPL
AFP-17	KKPTPASIPLFQVPEPVT
AFP-18	PLFQVPEPVTSCEAYEED
AFP-19	VTSCEAYEEDRETFMNKF
AFP-20	EDRETFMNKFIYEIARRH
AFP-21	KFIYEIARRHPFLYAPTI
AFP-22	RHPFLYAPTILLWAARYD
AFP-23	TILLWAARYDKIIPSCCK
AFP-24	YDKIIPSCCKAENAVECF
AFP-25	CKAENAVECFQTKAATVT
AFP-26	CFQTKAATVTKELRESSL
AFP-27	VTKELRESSLLNQHACAV
AFP-28	SLLNQHACAVMKNFGTRT
AFP-29	AVMKNFGTRTFQAITVTK
AFP-30	RTFQAITVTKLSQKFTKV
AFP-31	TKLSQKFTKVNFTEIQKL
AFP-32	KVNFTEIQKLVLDVAHVH
AFP-33	KLVLDVAHVHEHCCRGDV
AFP-34	VHEHCCRGDVLDCLQDGE
AFP-35	DVLDCLQDGEKIMSYICS
AFP-36	GEKIMSYICSQQDTLSNK
AFP-37	CSQQDTLSNKITECCKLT
AFP-38	NKITECCKLTTLERGQCI
AFP-39	LTTLERGQCIIHAENDEK
AFP-40	CIIHAENDEKPEGLSPNL
AFP-41	EKPEGLSPNLNRFLGDRD
AFP-42	NLNRFLGDRDFNQFSSGE
AFP-43	RDFNQFSSGEKNIFLASF
AFP-44	GEKNIFLASFVHEYSRRH
AFP-45	SFVHEYSRRHPQLAVSVI
AFP-46	RHPQLAVSVILRVAKGYQ
AFP-47	VILRVAKGYQELLEKCFQ
AFP-48	YQELLEKCFQTENPLECQ
AFP-49	FQTENPLECQDKGEEELQ
AFP-50	CQDKGEEELQKYIQESQA
AFP-51	LQKYIQESQALAKRSCGL
AFP-52	QALAKRSCGLFQKLGEYY
AFP-53	GLFQKLGEYYLQNAFLVA
AFP-54	YYLQNAFLVAYTKKAPQL
AFP-55	VAYTKKAPQLTSSELMAI
AFP-56	QLTSSELMAITRKMAATA
AFP-57	AITRKMAATAATCCQLSE
AFP-58	TAATCCQLSEDKLLACGE
AFP-59	SEDKLLACGEGAADIIIG
AFP-60	GEGAADIIIGHLCIRHEM
AFP-61	IGHLCIRHEMTPVNPGVG
AFP-62	EMTPVNPGVGQCCTSSYA
AFP-63	VGQCCTSSYANRRPCFSS
AFP-64	YANRRPCFSSLVVDETYV
AFP-65	SSLVVDETYVPPAFSDDK
AFP-66	YVPPAFSDDKFIFHKDLC
AFP-67	DKFIFHKDLCQAQGVALQ
AFP-68	LCQAQGVALQTMKQEFLI
AFP-69	LQTMKQEFLINLVKQKPQ
AFP-70	LINLVKQKPQITEEQLEA
AFP-71	PQITEEQLEAVIADFSGL
AFP-72	EAVIADFSGLLEKCCQGQ
AFP-73	GLLEKCCQGQEQEVCFAE
AFP-74	GQEQEVCFAEEGQKLISK
AFP-75	AEEGQKLISKTRAALGV
SALL-4-1	MSRRKQAKPQHINSEEDQ
SALL-4-2	PQHINSEEDQGEQQPQQQ
SALL-4-3	DQGEQQPQQQTPEFADAA
SALL-4-4	QQTPEFADAAPAAPAAGE
SALL-4-5	AAPAAPAAGELGAPVNHP
SALL-4-6	GELGAPVNHPGNDEVASE
SALL-4-7	HPGNDEVASEDEATVKRL
SALL-4-8	SEDEATVKRLRREETHVC
SALL-4-9	RLRREETHVCEKCCAEFF
SALL-4-10	VCEKCCAEFFSISEFLEH
SALL-4-11	FFSISEFLEHKKNCTKNP
SALL-4-12	EHKKNCTKNPPVLIMNDS
SALL-4-13	NPPVLIMNDSEGPVPSED
SALL-4-14	DSEGPVPSEDFSGAVLSH
SALL-4-15	EDFSGAVLSHQPTSPGSK
SALL-4-16	SHQPTSPGSKDCHRENGG
SALL-4-17	SKDCHRENGGSSEDMKEK
SALL-4-18	GGSSEDMKEKPDAESVVY
SALL-4-19	EKPDAESVVYLKTETALP
SALL-4-20	VYLKTETALPPTPQDISY
SALL-4-21	LPPTPQDISYLAKGKVAN
SALL-4-22	SYLAKGKVANTNVTLQAL
SALL-4-23	ANTNVTLQALRGTKVAVN
SALL-4-24	ALRGTKVAVNQRSADALP
SALL-4-25	VNQRSADALPAPVPGANS
SALL-4-26	LPAPVPGANSIPWVLEQI
SALL-4-27	NSIPWVLEQILCLQQQQL
SALL-4-28	QILCLQQQQLQQIQLTEQ
SALL-4-29	QLQQIQLTEQIRIQVNMW
SALL-4-30	EQIRIQVNMWASHALHSS
SALL-4-31	MWASHALHSSGAGADTLK
SALL-4-32	SSGAGADTLKTLGSHMSQ
SALL-4-33	LKTLGSHMSQQVSAAVAL
SALL-4-34	SQQVSAAVALLSQKAGSQ
SALL-4-35	ALLSQKAGSQGLSLDALK
SALL-4-36	SQGLSLDALKQAKLPHAN
SALL-4-37	LKQAKLPHANIPSATSSL
SALL-4-38	ANIPSATSSLSPGLAPFT
SALL-4-39	SLSPGLAPFTLKPDGTRV
SALL-4-40	FTLKPDGTRVLPNVMSRL
SALL-4-41	RVLPNVMSRLPSALLPQA
SALL-4-42	RLPSALLPQAPGSVLFQS
SALL-4-43	QAPGSVLFQSPFSTVALD
SALL-4-44	QSPFSTVALDTSKKGKGK
SALL-4-45	LDTSKKGKGKPPNISAVD
SALL-4-46	GKPPNISAVDVKPKDEAA
SALL-4-47	VDVKPKDEAALYKHKCKY
SALL-4-48	AALYKHKCKYCSKVFGTD
SALL-4-49	KYCSKVFGTDSSLQIHLR
SALL-4-50	TDSSLQIHLRSHTGERPF
SALL-4-51	LRSHTGERPFVCSVCGHR
SALL-4-52	PFVCSVCGHRFTTKGNLK
SALL-4-57	VAAGNGIPYALSVPDPID
SALL-4-58	YALSVPDPIDEPSLSLDS
SALL-4-59	IDEPSLSLDSKPVLVTTS
SALL-4-60	DSKPVLVTTSVGLPQNLS
SALL-4-61	TSVGLPQNLSSGTNPKDL
SALL-4-62	LSSGTNPKDLTGGSLPGD
SALL-4-63	DLTGGSLPGDLQPGPSPE
SALL-4-64	GDLQPGPSPESEGGPTLP
SALL-4-65	PESEGGPTLPGVGPNYNS
SALL-4-66	LPGVGPNYNSPRAGGFQG
SALL-4-67	NSPRAGGFQGSGTPEPGS
SALL-4-68	QGSGTPEPGSETLKLQQL
SALL-4-69	GSETLKLQQLVENIDKAT
SALL-4-70	QLVENIDKATTDPNECLI
SALL-4-71	ATTDPNECLICHRVLSCQ
SALL-4-72	LICHRVLSCQSSLKMHYR
SALL-4-73	CQSSLKMHYRTHTGERPF
SALL-4-74	YRTHTGERPFQCKICGRA
SALL-4-75	PFQCKICGRAFSTKGNLK
SALL-4-76	RAFSTKGNLKTHLGVHRT
SALL-4-77	LKTHLGVHRTNTSIKTQH
SALL-4-78	RTNTSIKTQHSCPICQKK
SALL-4-79	QHSCPICQKKFTNAVMLQ
SALL-4-80	KKFTNAVMLQQHIRMHMG
SALL-4-81	LQQHIRMHMGGQIPNTPL
SALL-4-82	MGGQIPNTPLPENPCDFT
SALL-4-83	PLPENPCDFTGSEPMTVG
SALL-4-84	FTGSEPMTVGENGSTGAI
SALL-4-85	VGENGSTGAICHDDVIES
SALL-4-86	AICHDDVIESIDVEEVSS
SALL-4-87	ESIDVEEVSSQEAPSSSS
SALL-4-88	SSQEAPSSSSKVPTPLPS
SALL-4-89	SSKVPTPLPSIHSASPTL
SALL-4-90	PSIHSASPTLGFAMMASL
SALL-4-91	TLGFAMMASLDAPGKVGP
SALL-4-92	SLDAPGKVGPAPFNLQRQ
SALL-4-93	GPAPFNLQRQGSRENGSV
SALL-4-94	RQGSRENGSVESDGLTND
SALL-4-95	SVESDGLTNDSSSLMGDQ
SALL-4-96	NDSSSLMGDQEYQSRSPD
SALL-4-97	DQEYQSRSPDILETTSFQ
SALL-4-98	PDILETTSFQALSPANSQ
SALL-4-99	FQALSPANSQAESIKSKS
SALL-4-100	SQAESIKSKSPDAGSKAE
SALL-4-101	KSPDAGSKAESSENSRTE
SALL-4-102	AESSENSRTEMEGRSSLP
SALL-4-103	TEMEGRSSLPSTFIRAPP
SALL-4-104	LPSTFIRAPPTYVKVEVP
SALL-4-105	PPTYVKVEVPGTFVGPST
SALL-4-106	VPGTFVGPSTLSPGMTPL
SALL-4-107	STLSPGMTPLLAAQPRRQ
SALL-4-108	PLLAAQPRRQAKQHGCTR
SALL-4-109	RQAKQHGCTRCGKNFSSA
SALL-4-110	TRCGKNFSSASALQIHER
SALL-4-111	SASALQIHERTHTGEKPF
SALL-4-112	ERTHTGEKPFVCNICGRA
SALL-4-113	PFVCNICGRAFTTKGNLK
SALL-4-114	RAFTTKGNLKVHYMTHGA
SALL-4-115	LKVHYMTHGANNNSARRG
SALL-4-116	GANNNSARRGRKLAIENT
SALL-4-117	RGRKLAIENTMALLGTDG
SALL-4-118	NTMALLGTDGKRVSEIFP
SALL-4-119	DGKRVSEIFPKEILAPSV
SALL-4-120	FPKEILAPSVNVDPVVWN
SALL-4-121	SVNVDPVVWNQYTSMLNG
SALL-4-122	WNQYTSMLNGGLAVKTNE
SALL-4-123	NGGLAVKTNEISVIQSGG
SALL-4-124	NEISVIQSGGVPTLPVSL
SALL-4-125	GGVPTLPVSLGATSVVNN
SALL-4-126	SLGATSVVNNATVSKMDG
SALL-4-127	NNATVSKMDGSQSGISAD
SALL-4-128	DGSQSGISADVEKPSATD
SALL-4-129	ADVEKPSATDGVPKHQFP
SALL-4-130	TDGVPKHQFPHFLEENKI
SALL-4-131	FPHFLEENKIAVS
MAGE-A3-1	MPLEQRSQHCKPEEGLEA
MAGE-A3-2	HCKPEEGLEARGEALGLV
MAGE-A3-3	EARGEALGLVGAQAPATE
MAGE-A3-4	LVGAQAPATEEQEAASSS
MAGE-A3-5	TEEQEAASSSSTLVEVTL
MAGE-A3-6	SSSTLVEVTLGEVPAAES
MAGE-A3-7	TLGEVPAAESPDPPQSPQ
MAGE-A3-8	ESPDPPQSPQGASSLPTT
MAGE-A3-9	PQGASSLPTTMNYPLWSQ
MAGE-A3-10	TTMNYPLWSQSYEDSSNQ
MAGE-A3-11	SQSYEDSSNQEEEGPSTF
MAGE-A3-12	NQEEEGPSTFPDLESEFQ
MAGE-A3-13	TFPDLESEFQAALSRKVA
MAGE-A3-14	FQAALSRKVAELVHFLLL
MAGE-A3-15	VAELVHFLLLKYRAREPV
MAGE-A3-16	LLKYRAREPVTKAEMLGS
MAGE-A3-17	PVTKAEMLGSVVGNWQYF
MAGE-A3-18	GSVVGNWQYFFPVIFSKA
MAGE-A3-19	YFFPVIFSKASSSLQLVF
MAGE-A3-20	KASSSLQLVFGIELMEVD
MAGE-A3-21	VFGIELMEVDPIGHLYIF
MAGE-A3-22	VDPIGHLYIFATCLGLSY
MAGE-A3-23	IFATCLGLSYDGLLGDNQ
MAGE-A3-24	SYDGLLGDNQIMPKAGLL
MAGE-A3-25	NQIMPKAGLLIIVLAIIA
MAGE-A3-26	LLIIVLAIIAREGDCAPE
MAGE-A3-27	IAREGDCAPEEKIWEELS
MAGE-A3-28	PEEKIWEELSVLEVFEGR
MAGE-A3-29	LSVLEVFEGREDSILGDP
MAGE-A3-30	GREDSILGDPKKLLTQHF
MAGE-A3-31	DPKKLLTQHFVQENYLEY
MAGE-A3-32	HFVQENYLEYRQVPGSDP
MAGE-A3-33	EYRQVPGSDPACYEFLWG
MAGE-A3-34	DPACYEFLWGPRALVETS
MAGE-A3-35	WGPRALVETSYVKVLHHM
MAGE-A3-36	TSYVKVLHHMVKISGGPH
MAGE-A3-37	HMVKISGGPHISYPPLHE
MAGE-A3-38	PHISYPPLHEWVLREGEE
MAGE-A3-39	HEWVLREGEE
MAGE-A1-1	MSLEQRSLHCKPEEALEA
MAGE-A1-2	HCKPEEALEAQQEALGLV
MAGE-A1-3	EAQQEALGLVCVQAATSS
MAGE-A1-4	LVCVQAATSSSSPLVLGT
MAGE-A1-5	SSSSPLVLGTLEEVPTAG
MAGE-A1-6	GTLEEVPTAGSTDPPQSP
MAGE-A1-7	AGSTDPPQSPQGASAFPT
MAGE-A1-8	SPQGASAFPTTINFTRQR
MAGE-A1-9	PTTINFTRQRQPSEGSSS
MAGE-A1-10	QRQPSEGSSSREEEGPST
MAGE-A1-11	SSREEEGPSTSCILESLF
MAGE-A1-12	STSCILESLFRAVITKKV
MAGE-A1-13	LFRAVITKKVADLVGFLL
MAGE-A1-14	KVADLVGFLLLKYRAREP
MAGE-A1-15	LLLKYRAREPVTKAEMLE
MAGE-A1-16	EPVTKAEMLESVIKNYKH
MAGE-A1-17	LESVIKNYKHCFPEIFGK
MAGE-A1-18	KHCFPEIFGKASESLQLV
MAGE-A1-19	GKASESLQLVFGIDVKEA
MAGE-A1-20	LVFGIDVKEADPTGHSYV
MAGE-A1-21	EADPTGHSYVLVTCLGLS
MAGE-A1-22	YVLVTCLGLSYDGLLGDN
MAGE-A1-23	LSYDGLLGDNQIMPKTGF
MAGE-A1-24	DNQIMPKTGFLIIVLVMA
MAGE-A1-25	GFLIIVLVMAMEGGHAPE
MAGE-A1-26	MAMEGGHAPEEEIWEELS
MAGE-A1-27	PEEEIWEELSVMEVYDGR
MAGE-A1-28	LSVMEVYDGREHSAYGEP
MAGE-A1-29	GREHSAYGEPRKLLTQDL
MAGE-A1-30	EPRKLLTQDLVQEKYLEY
MAGE-A1-31	DLVQEKYLEYRQVPDSDP
MAGE-A1-32	EYRQVPDSDPARYEFLWG
MAGE-A1-33	DPARYEFLWGPRALAETS
MAGE-A1-34	WGPRALAETSYVKVLEYV
MAGE-A1-35	TSYVKVLEYVIKVSARVR
MAGE-A1-36	YVIKVSARVRFFFPSLRE
MAGE-A1-37	VRFFFPSLREAALREEEE
MAGE-A1-38	REAALREEEEGVMSLEQR
MAGE-A1-39	EEGVMSLEQRSLHCKPEE
NY-ESO-1-1	MQAEGRGTGGSTGDADGP
NY-ESO-1-2	GGSTGDADGPGGPGIPDG
NY-ESO-1-3	GPGGPGIPDGPGGNAGGP
NY-ESO-1-4	DGPGGNAGGPGEAGATGG
NY-ESO-1-5	GPGEAGATGGRGPRGAGA
NY-ESO-1-6	GGRGPRGAGAARASGPGG
NY-ESO-1-7	GAARASGPGGGAPRGPHG
NY-ESO-1-8	GGGAPRGPHGGAASGLNG
NY-ESO-1-9	HGGAASGLNGCCRCGARG
NY-ESO-1-10	NGCCRCGARGPESRLLEF
NY-ESO-1-11	RGPESRLLEFYLAMPFAT
NY-ESO-1-12	EFYLAMPFATPMEAELAR
NY-ESO-1-13	ATPMEAELARRSLAQDAP
NY-ESO-1-14	ARRSLAQDAPPLPVPGVL
NY-ESO-1-15	APPLPVPGVLLKEFTVSG
NY-ESO-1-16	VLLKEFTVSGNILTIRLT
NY-ESO-1-17	SGNILTIRLTAADHRQLQ
NY-ESO-1-18	LTAADHRQLQLSISSCLQ
NY-ESO-1-19	LQLSISSCLQQLSLLMWI
NY-ESO-1-20	LQQLSLLMWITQCFLPVF
NY-ESO-1-21	WITQCFLPVFLAQPPSGQ
NY-ESO-1-22	VFLAQPPSGQRRMQAEGR
NY-ESO-1-23	GQRRMQAEGRGTGGSTGD
SSX-2-1	MNGDDAFARRPTVGAQIP
SSX-2-2	RRPTVGAQIPEKIQKAFD
SSX-2-3	IPEKIQKAFDDIAKYFSK
SSX-2-4	FDDIAKYFSKEEWEKMKA
SSX-2-5	SKEEWEKMKASEKIFYVY
SSX-2-6	KASEKIFYVYMKRKYEAM
SSX-2-7	VYMKRKYEAMTKLGFKAT
SSX-2-8	AMTKLGFKATLPPFMCNK
SSX-2-9	ATLPPFMCNKRAEDFQGN
SSX-2-10	NKRAEDFQGNDLDNDPNR
SSX-2-11	GNDLDNDPNRGNQVERPQ
SSX-2-12	NRGNQVERPQMTFGRLQG
SSX-2-13	PQMTFGRLQGISPKIMPK
SSX-2-14	QGISPKIMPKKPAEEGND
SSX-2-15	PKKPAEEGNDSEEVPEAS
SSX-2-16	NDSEEVPEASGPQNDGKE
SSX-2-17	ASGPQNDGKELCPPGKPT
SSX-2-18	KELCPPGKPTTSEKIHER
SSX-2-19	PTTSEKIHERSGNREAQE
SSX-2-20	ERSGNREAQEKEERRGTA
SSX-2-21	QEKEERRGTAHRWSSQNT
SSX-2-22	TAHRWSSQNTHNIGRFSL
SSX-2-23	NTHNIGRFSLSTSMGAVH
SSX-2-24	SLSTSMGAVHGTPKTITH
SSX-2-25	VHGTPKTITHNRDPKGGN
SSX-2-26	THNRDPKGGNMPGPTDCV
SSX-2-27	GNMPGPTDCVRENSW

The design of the nine pools: Pool 1: SALL4 _1-45,_ Pool 2: SALL4 _46-90_, Pool 3: SALL4 _91-131_, Pool 4: MAGE-A1 _1-39_, Pool 5: MAGE-A3 _1-39_, Pool 6: NY-ESO-1 _1-23_, Pool 7: SSX2 _1-27_, Pool 8: AFP _1-40_, Pool 9: AFP _41-75._

### Human IFN-γ ELISPOT assay

IFN-γ ELISPOT assay were performed as described (14). A total of 250,000 PBMCs with 8 μg/mL peptide per well containing RPMI 1640 medium with 10% FCS were used in a standard human IFN-γ ELISPOT assay. In brief, assays were carried out in 96-well MultiScreen filter plates (Millipore) coated with 15 mg/mL anti–IFN-γ mAb (1-DIK; Mabtech). Phytohaemagglutinin (10 μg/mL) was used as a positive control. Plates were incubated for 16–18 h. The plate was washed 5 times, and biotin-conjugated anti-human IFN-γ Ab (Mabtech, Nacka, Sweden) was added and reacted for 2 h. After washing the plate 5 times, streptavidin-ALP (Mabtech, Nacka, Sweden) was added and reacted for 1 h. Finally, newly prepared NBT/BCIP solution (Bio-Rad, Hercules, CA) was added for colour development after washing. The reaction was stopped by washing with distilled water, and the plate was dried at room temperature. Spot enumeration was performed with a CTL ELISPOT reader system (Cellular Technology Ltd, S6 Universal, America). The number of specific spots was determined by subtracting the number of spots in the absence of antigen from the number in its presence, and the results were expressed as spot-forming units (SFUs) per 10^6^ PBMCs. Responses were regarded as positive if the results were at least three times the mean of the negative control wells and above 25 SFUs/10^6^ PBMCs. If background wells were 25 SFUs/10^6^ PBMCs or positive control wells were negative, the results were excluded from further analysis.

### Generation of tumour antigen specific T-cell lines

Totally, 57 antigen-specific T cell lines from 42 subjects were generated. Cells stimulated by peptides were collected after culturation for 16-18 hours in the ELISPOT assay, and then were used for the generation of T cell lines. The cells were grown in 96-well plates. Short-term T cell lines were grown for 10-14 days in AIM-V + 10% human AB serum (Invitrogen, Carlsbad, CA) supplemented with 100 μg/mL (final concentration) interleukin (IL)-2 (R&D Systems, Minneapolis, MN).

### Flow cytometry

The generated short-term T cell lines were stimulated with mixed TAAs for 4 hours and cells without stimulation were used as negative controls. Then, cells were stained with LIVE/DEAD Fixable Aqua Dead Cell Stain Kit (Thermo Fisher Scientific) and surface markers, including CD3-AF700 (Bio Legend), CD4-FITC (BD Biosciences), CD8-APC-H7 (BD Biosciences), PD1-BV650 (BD Biosciences), and Tim3-BV421 (Bio Legend), fixed with 1 × CellFix solution (BD Biosciences) and acquired immediately on a BD LSR Fortessa. Fluorescence minus one (FMO) controls were applied accordingly in order to properly position gates.

In the validation cohort, 60 samples from 26 HCC patients were thawed and rested overnight. These cells were stained with LIVE/DEAD Fixable Aqua Dead Cell Stain Kit (Thermo Fisher Scientific) and surface markers, including CD3-BV786 (Bio Legend), CD4-BV711 (Bio Legend), CD8-Percp-cy5.5 (BD Biosciences), and CD39-PE-CF594 (Bio Legend), fixed with 1 × CellFix solution (BD Biosciences), and acquired immediately on a BD LSR Fortessa. Flow data were analyzed by FlowJo V.10.0.

### Statistical analysis

Continuous variables are expressed as the mean ± standard deviation (SD). Statistical analysis of the data was performed using the χ^2^ test for constituent ratio analysis. Two-tailed Student’s t tests were used to compare parametric continuous data, and the Mann-Whitney U test was used when data were not normally distributed. Statistical significance was set at P < 0.05. Analyses were performed with SPSS software v25 (IBM, New York, USA), and graphs were constructed with GraphPad Prism 8.0 (GraphPad software Inc).

## Results

### Patient characteristics

The epidemiological, pathological, and clinical parameters of the enrolled patients in the present study are summarized in **Table 2**. The demographic and oncological characteristics between the patients at baseline (BF), and at 1 week (1W), and after 4 weeks (4W) did not show significant difference. At 1W, values of indexes of liver injury and inflammation increased, including those of the white blood cell count (WBC), glutamic-pyruvic transaminase (ALT), and glutamic oxalacetic transaminase (AST). Indicators of the basic status of the patient decreased, including hemoglobin (HGB) and albumin (ALB). At 4W, the levels of tumor biomarkers were lower than those of patients at BF (**Table 2A**).

**Table 2A:** Characteristics of enrolled individuals without VI/M and achieved curative therapy before and after ablation.

Characteristic	BF (n=57)	AF (n=80)		P_a_	P_b_
		1W (n=49)	4W (n=31)		
Gender (male/female)	44/13	38/11	25/6	0.965	0.707
Age	55.32 ± 9.06	56.46 ± 10.19	54.27 ± 9.08	0.47	0.599
Pathogeny (HBV/other)	51/6	46/3	29/2	0.5	0.805
Liver cirrhosis (no/compensated/decompensated)	9/32/16	5/29/15	5/17/9	0.697	0.993
Differentiation (well/moderate/poor/ND)	3/5/8/41	4/5/8/32	2/5/5/19	/	/
BCLC stage (0/A/B)	7/42/8	8/36/5	4/23/4	0.733	0.987
WBC (10^9^/L)	4.97 ± 2.02	6.59 ± 2.79	4.92 ± 1.83	**0.003**	0.985
HGB (g/L)	142.55 ± 17.45	129.67 ± 18.53	143.21 ± 19.16	**0.001**	0.839
PLT (10^9^/L)	136.75 ± 71.36	153.63 ± 67.22	147.90 ± 60.60	0.168	0.344
ALT (U/L)	31.89 ± 23.35	138.84 ± 117.44	29.36 ± 11.74	**0.000**	0.674
AST (U/L)	31.98 ± 16.02	84.45 ± 65.08	30.00 ± 9.08	**0.000**	0.716
TBiL (μmmol/L)	17.74 ± 8.96	19.96 ± 12.21	17.80 ± 8.13	0.454	0.866
ALB (g/L)	39.86 ± 4.65	35.40 ± 4.13	41.38 ± 4.66	**0.000**	0.2
PT (s)	12.49 ± 1.48	13.00 ± 1.28	12.07 ± 0.96	0.062	0.324
PTA (%)	86.39 ± 13.71	80.88 ± 11.08	89.87 ± 11.24	0.07	0.347
AFP (ng/mL)	2098.05 ± 13144.41	96.29 ± 206.93	44.27 ± 168.23	0.714	**0.005**
PIVKA-II (mAU/mL)	2110.42 ± 9312.93	98.19 ± 181.42	34.04 ± 20.92	0.18	**0.000**

Bold font indicates statistical significance of P values.

Among the 28 patients who were enrolled in the matching analysis, two patients were classified as BCLC-0, 23 as BCLC-A, and 3 as BCLC-B stage. Similarly, the level of WBC, ALT, AST, and prothrombin time (PT) increased, and the level of HGB, ALB and prothrombin activity (PTA) decreased significantly 1W after ablation **Table 2B**. In the matched analysis of the 1W:4W cohort, most patients had BCLC-A grade HCC (18/22, 81.82%), and there were 2 patients each with stage 0 and stage B HCC. Four weeks after ablation, the transformation from BF to 1W was reversed, and the level of protein induced by vitamin K absence or antagonist-II (PIVKA-II) was significantly reduced **Table 2C**.

**Table 2B:** Characteristics of patients in the BF and 1W cohort.

Characteristic (n=28)	BF	1W	P
Gender (male/female)	23/5		
Age	54.39 ± 10.10		
Pathogeny (HBV/other)	27/1		
Liver cirrhosis (no/compensated/decompensated)	6/15/7		
Differentiation (well/moderate/poor/ND)	2/3/5/18		
BCLC stage (0/A/B)	2/23/3		
WBC (10^9^/L)	5.64 ± 2.30	7.45 ± 2.58	**0.008**
HGB (g/L)	146.79 ± 16.67	133.46 ± 15.80	**0.006**
PLT (10^9^/L)	137.71 ± 53.93	160 ± 56.33	0.177
ALT (U/L)	32.23 ± 23.52	166.46 ± 138.31	**0.000**
AST (U/L)	30.36 ± 17.49	81.29 ± 67.12	**0.000**
TBiL (μmmol/L)	15.75 ± 6.28	18.85 ± 12.95	0.653
ALB (g/L)	40.57 ± 4.41	35.46 ± 4.07	**0.000**
PT (s)	11.99 ± 0.94	13.01 ± 1.34	**0.028**
PTA (%)	90.80 ± 11.30	80.63 ± 12.52	**0.026**
AFP (ng/mL)	341.91 ± 1036.78	114.45 ± 241.05	0.427
PIVKA-II (mAU/mL)	189.20 ± 279.96	73.29 ± 64.94	0.343

Bold font indicates statistical significance of P values.

**Table 2C:** Characteristics of patients in the 1W and 4W cohort.

Characteristic (n=22)	1W	4W	P
Gender (male/female)	17/5		
Age	55.14 ± 8.79		
Pathogeny (HBV/other)	21/1		
Liver cirrhosis (no/compensated/decompensated)	2/12/8		
Differentiation (well/moderate/poor/ND)	3/3/3/13		
BCLC stage (0/A/B)	2/18/2		
WBC (10^9^/L)	6.77 ± 3.10	4.81 ± 1.64	**0.042**
HGB (g/L)	128.95 ± 20.07	142.48 ± 19.60	**0.03**
PLT (10^9^/L)	167 ± 70.11	154.14 ± 67.91	0.473
ALT (U/L)	131.14 ± 113.37	29.45 ± 11.67	**0.000**
AST (U/L)	69.77 ± 56.97	30.75 ± 10.05	**0.001**
TBiL (μmmol/L)	16.12 ± 7.60	17.57 ± 7.44	0.641
ALB (g/L)	34.67 ± 4.18	41.31 ± 4.98	**0.000**
PT (s)	12.71 ± 1.12	11.89 ± 0.90	**0.032**
PTA (%)	82.93 ± 10.72	91.81 ±10.57	**0.026**
AFP (ng/mL)	113.17 ± 260.11	10.78 ± 21.47	0.124
PIVKA-II (mAU/mL)	71.06 ± 63.61	37.29 ± 23.87	**0.018**

Bold font indicates statistical significance of P values.

### Dynamic response of TAA-specific T cells in patients at BF, 1W and 4W after ablation

**Figure 2:**
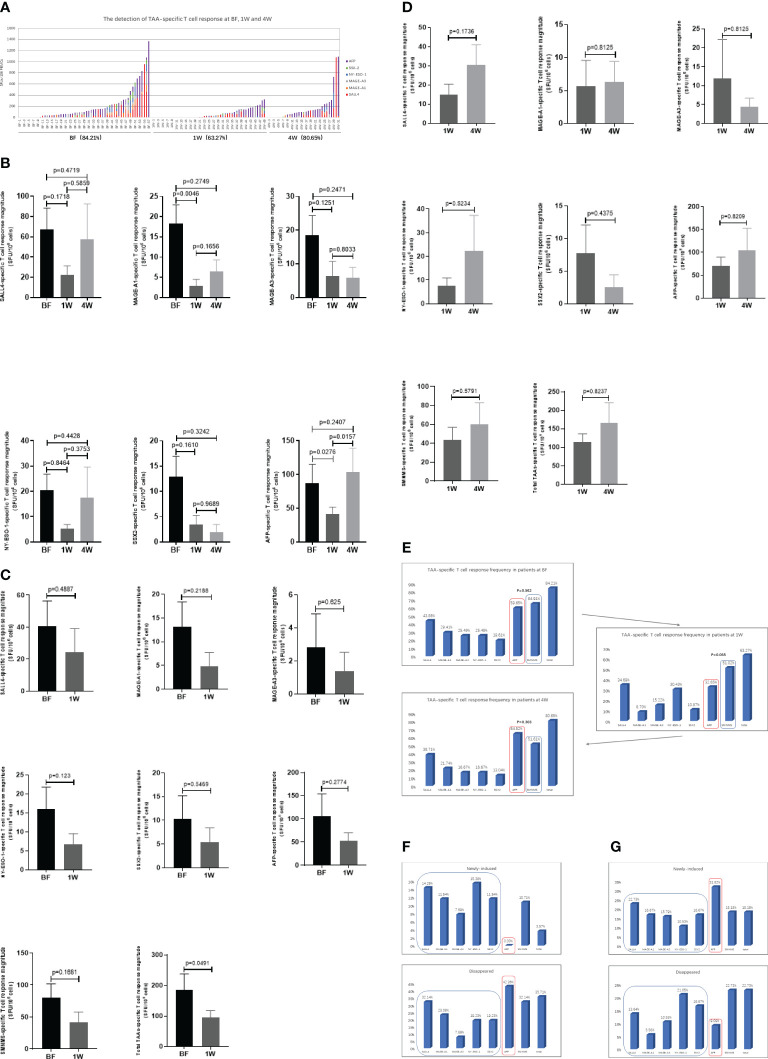
The detection of TAA-specific T cell responses in HCC patients at before (BF), 1week (1W) and 4 weeks (4W) after ablation by IFN-γ ELISPOT assay. **(A)** The distribution of TAA-specific T cell responses specific to AFP (purple), SALL4 (red), MAGE-A3 (grey), MAGE-A1 (orange), NY-ESO-1 (blue) and SSX2 (green) in HCC patients at BF (n=57), 1W (n=49), and 4W (n=31). The magnitude of T cell response was evaluated with SFUs/106 PBMCs in vertical coordinates (y axis), and the groups were labelled in horizontal ordinate (x axis). **(B)** The TAA-specific T cell response magnitude was analyzed between patients at BF (n=57), 1W (n=49), and 4W (n=31). Values were compared by Mann-Whitney U-test. **(C)** The matching analysis of TAA-specific T cell response magnitude between BF and 1W (n=28). Values were compared by paired non-parametric test. **(D)** The matching analysis of TAA-specific T cell response magnitude between 1W and 4W (n=22). Values were compared by paired non-parametric test. **(E)** The TAA-specific T cell immune response frequency and the appearance of the immune profile at BF (n=57), 1W (n=49), and 4W (n=31). Values were compared by chi-squared test. **(F)** The alteration (newly-induced and disappeared) of TAA-specific T cell immune response frequency and the appearance of the immune profile from BF to 1W (n=28). **(G)** The alteration (newly-induced and disappeared) of TAA-specific T cell immune response frequency and the appearance of the immune profile from 1W to 4W (n=22).

All *ex vivo* samples underwent direct testing using the IFN-γ ELISPOT assay. As shown in **Figure 2A**, the distribution of the specific T cell immune response against each TAA was depicted. A positive TAA-specific T cell response was observed in 84.21% (48/57), 63.27% (31/49), and 80.65% (25/31) of HCC patients at BF, 1W, and 4W, respectively. No significant changes were observed between these patients.

The magnitude of the TAA-specific T cell responses was determined by the frequency of T cells. The response magnitudes in patients at BF, 1W, and 4W are shown in **Figure 2B**. Among these TAA-specific T cell responses, only MAGE-A1 and AFP-specific T cell response magnitude in the 1W group was lower than that of patients in the BF group (MAGE-A1: 18.33 ± 4.6 vs. 2.835 ± 1.648 SFUs/10^6^ cells, P=0.0046; AFP: 87.53 ± 27.6 vs. 40.89 ± 10.73 SFUs/10^6^ cells, P=0.0276). In addition, the magnitude of the AFP-specific T cell response of patients in the 4W group (103.9 ± 35.06 SFUs/10^6^ cells) was stronger than that of patients in the 1W group (40.89 ± 10.73 SFUs/10^6^ cells) (P=0.0157). Furthermore, most TAA-specific T cell numbers did not show significant differences between patients at the three time points. To further examine the effect of ablation on TAA-specific T cells, an analysis was performed in matching patients at BF:1W and 1W: 4W data. Similarly, no significant alteration was found (**Figures 2C, D**).

The T cell response frequency against each TAA was analyzed and compared in terms of the presence of two distinct TAA-specific immune response profiles in HCC (14). In patients at BF, the AFP-specific T cell response frequency was 59.65% (34/57), which was similar to the SMNMS-specific T cell response, 64.91% (37/57), P=0.562. Interestingly, for patients at 1W post-ablation, the SMNMS-specific T cell response frequency (51.02%, 25/49) showed a tendency to be higher than that of the AFP-specific T cell response (32.65%, 16/49) (P=0.065). However, after 4W, the frequency of the AFP-specific T cell response was higher than the frequency of the SMNMS-specific T cell response (**Figure 2E**).

Furthermore, on comparing ablation-treated patients with matched BF samples, interesting results were obtained. Comparing the BF to the 1W response, none of the patients presented a newly induced AFP-specific T cell response, however all the patients with newly induced T-cell immune response achieved a SMNMS-specific T cell response, ranging from 7.69% (2/26) to 15.38% (4/26). For patients whose AFP-specific T cell response disappeared 1W after ablation accounted for 42.86% (12/28), with the highest frequency (**Figure 2F**). However, from 1W to 4W, patients with newly induced TAA-specific T cell response was the highest against AFP, 31.82% (7/22), followed by the SMNMS-specific T cell response: SALL4, MAGE-A1, SSX2, MAGE-A3, and NY-ESO-1. Also, among the patients with T cell response that disappeared, the frequency of AFP-specific T cell response (9.09%, 2/22) was low relative to the SMNMS-specific T cell response (22.73%, 5/22).

Taken together, the magnitude of TAA-specific T cell response was not significantly affected by ablation treatment, although the immune response profile improved 1W after ablation, this immune response profile was absent after 4W.

### Effect of the transformation of the TAA-specific T cell response after ablation on the prognosis of patients

**Figure 3:**
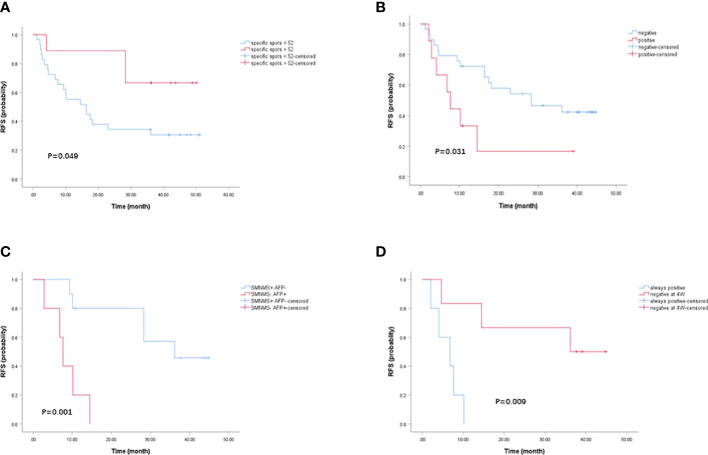
Kaplan-Meier curves of HCC recurrence-free survival. **(A)** Kaplan-Meier curves indicating the relationship between month after ablation and HCC recurrence-free survival rate were grouped by the median number of SMNMS-specific T cells detected by IFN-γ ELISPOT assay in patients with positive SMNMS-specific T cell response at 1W. **(B)** Kaplan-Meier curves indicating the relationship between month after ablation and HCC recurrence-free survival rate were grouped by the presence of AFP-specific T cell response detected by IFN-γ ELISPOT assay at 1W. **(C)** Kaplan-Meier curves indicating the relationship between month after ablation and HCC recurrence-free survival rate were depicted between patients with “SMNMS+ AFP-” specific T cell response and patients with “SMNMS- AFP+” specific T cell detected by IFN-γ ELISPOT assay at 1W. **(D)** Kaplan-Meier curves indicating the relationship between month after ablation and HCC recurrence-free survival rate were grouped by the presence of AFP-specific T cell response detected by IFN-γ ELISPOT assay from 1W to 4W.

To examine the effects of the transformation of the TAA-specific T cell response after ablation on the prognosis of patients with HCC, we analyzed the relationship between the immune response and recurrence-free survival (RFS) of patients with HCC after ablation. First, we divided patients into two groups with high (above median) and low (below median) specific spots detected by the IFN-γ ELISPOT assay in patients with a positive SMNMS-specific T cell response at 1W. We found that a high number of SMNMS-specific T cells after HCC treatment correlated with the RFS (P=0.049) (**Figure 3A**). Conversely, a marked difference between the groups was emphasized when patients were divided according to the presence or absence of an AFP-specific T cell response (P=0.031) (**Figure 3B**). As shown in **Figures 2E, F**, there was a difference in the TAA-specific T cell response profile between the BF, 1W and 4W groups. In the 1W post-ablation group, the presence of this TAA-specific T cell response profile was advantageous for patients (14). Furthermore, we found that patients with “SMNMS+ AFP-” specific T cell response achieved a significantly higher RFS than those with “SMNMS- AFP+” specific T cell response at 1W (P=0.001) (**Figure 3C**). Unfortunately, as shown in **Figure 2G**, this improved TAA-specific T cell response profile observed at 1W could not be maintained until week-4. The frequency of patients whose AFP-specific T cell immune response disappeared at 4W after ablation was relatively low. Unfortunately, the presence of an AFP-specific T cell response at 4W post-ablation indicated a rapid tumor progression (P=0.009, **Figure 3D**).

### Phenotypic analysis of TAA-specific T cells before and after ablation

**Figure 4:**
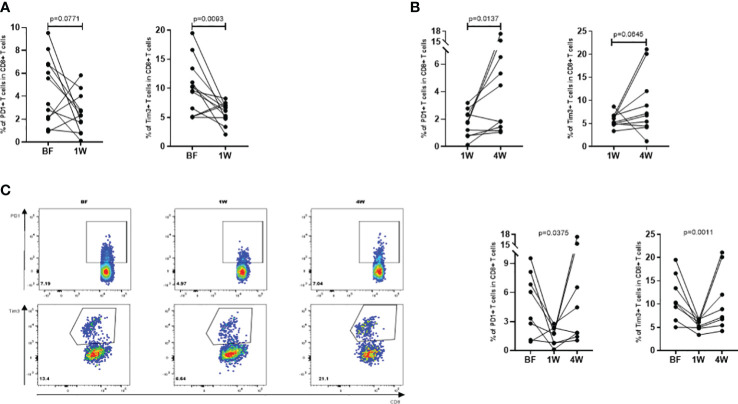
Phenotypic analysis of the TAA-specific T cells at BF, 1W and 4W. **(A)** The BF: 1W matching analysis of the PD1+ and Tim3+ T cells in TAA-specific CD8+ T cells using the culturation of T cell (n=12). **(B)** The 1W: 4W matching analysis of the PD1+ and Tim3+ T cells in TAA-specific CD8+ T cells using the culturation of T cell (n=10). **(C)** The BF: 1W: 4W matching analysis of the PD1+ and Tim3+ T cells in TAA-specific CD8+ T cells using the culturation of T cell (n=10). Values were compared by paired non-parametric test.

Since T cell function is restricted by immune checkpoints, to identify the relationship between TAA-specific T cell response and HCC recurrence, we examined the expression of the exhaustion markers represented by PD1 and Tim3 on TAA-specific CD8+ T cells. Comparing pre-ablation status BF with the 1W after ablation, the percentage of CD8+PD1+ T cells decreased from 4.6 ± 0.84 to 2.49 ± 0.49, with an evident decreasing trend (P=0.0771). Furthermore, there was a significant decrease in the percentage of CD8+Tim3+ T cells at 1W (5.78 ± 0.51) compared to BF (10.14 ± 1.32) (P=0.0093) (**Figure 4A**). These results indicated that these T cells were in a more powerful functional state at 1W. However, in the analysis of immune checkpoint expression between 1W and 4W, the percentage of CD8+PD1+ specific T cells increased from 1.70 ± 0.31 to 5.63 ± 1.89 (P=0.0137) and CD8+Tim3+ specific T cells increased from 5.81 ± 0.46 to 9.12 ± 2.12 (P=0.0645) (**Figure 4B**), indicating a restriction of the antitumor capacity of these T cells. Interestingly, we confirmed these results in the dynamic cohort of patients at three time points at BF, 1W and 4W after receiving ablative therapy (**Figure 4C**). The characteristics of these patients are shown in (**Table 2D**).

**Table 2D:** Characteristics of patients in the BF, 1W, and 4W cohort.

Characteristic (n=16)	BF	1W	4W	P_a_	P_b_	
Gender (male/female)	11/5					
Age	55.13 ± 8.25					
Pathogeny (HBV/other)	16/0					
Liver cirrhosis (no/compensated/decompensated)	2/9/5					
Differentiation (well/moderate/poor/ND)	1/2/3/10					
BCLC stage (0/A/B)	2/12/2					
WBC (10^9^/L)	5.76 ± 2.12	7.13 ± 2.78	5.00 ± 2.21	0.146		0.253
HGB (g/L)	145.79 ± 17.70	131.31 ± 14.96	143.69 ± 17.30	**0.023**		0.771
PLT (10^9^/L)	153.36 ± 58.85	177.31 ± 58.56	156 ± 67.43	0.299		0.884
ALT (U/L)	29.17 ± 13.27	129.43 ± 114.29	29.13 ± 12.69	**0.000**		0.883
AST (U/L)	28.33 ± 17.59	50.13 ± 26.65	28.53 ± 7.99	**0.001**		0.203
TBiL (μmmol/L)	14.85 ± 4.30	13.98 ± 4.66	15.05 ± 4.22	0.516		0.961
ALB (g/L)	41.61 ± 4.92	34.82 ± 3.92	41.88 ± 4.85	**0.001**		0.751
PT (s)	11.77 ± 0.88	12.64 ± 1.32	11.94 ± 0.88	0.232		0.897
PTA (%)	93.53 ± 11.61	83.67 ± 12.73	91.18 ± 9.69	0.179		0.897
AFP (ng/mL)	469.88 ± 1361.61	146.87 ± 295.46	11.31 ± 24.27	0.738		0.097
PIVKA-II (mAU/mL)	148 ± 164.35	76.42 ± 71.41	36.27 ± 21.16	0.44		**0.019**

VI/M, vascular invasion/metastasis. HBV, hepatitis B virus. WBC, White Blood Cell. HGB, hemoglobin. PLT, platelet. ALT, alanine aminotransferase. AST, aspartate aminotransferase. TBil, total bilirubin. ALB, albumin. PT, prothrombin time. PTA, prothrombin activity. AFP, alpha-fetoprotein. PIVKA-II, protein induced by vitamin K absence or antagonist-II.

Data were expressed as mean ± SD. _a_: The data of the 1W group was compared with the BF group, P<0.05. _b_: The data of the 4W group was compared with the BF group, P<0.05.

Bold font indicates statistical significance of P values.

### Analysis of tumor-specific T cells in the peripheral circulation defined by CD8+CD39+ T cells

**Figure 5:**
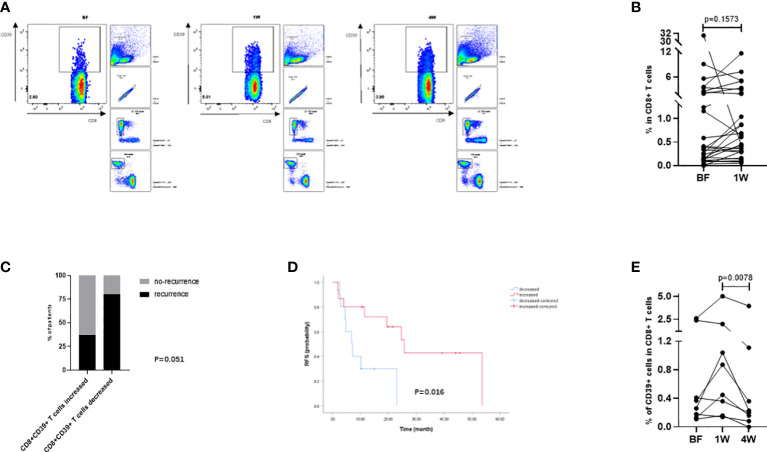
Analysis of tumor-specific T cells in peripheral circulation defined by CD8+CD39+ T cells. **(A)** The gating strategy of CD8+CD39+ T cells in flowcytometry analysis. **(B)** The compared BF: 1W matching analysis of the CD8+CD39+ T cells (n=26). **(C)** The changes of CD8+CD39+ T cells at 1W were compared with those at BF according to the prognosis of patients one year after ablation (n=26). **(D)** Kaplan-Meier curves indicating the relationship between month after ablation and HCC recurrence-free survival rate were grouped by the changes of CD8+CD39+ T cells from BF to 1W (n=26). **(E)** The BF: 1W: 4W matching analysis of the CD8+CD39+ T cells (n=8). Values were compared by paired non-parametric test.

To further confirm the above findings in TAA-specific T cells, specific T cells defined by CD8+CD39+ T cells from PBMC samples (**Figure 5A**) from 26 patients (**Table 3**) were isolated and detected by flow cytometry. At 1W after ablation, double positive CD8+CD39+ T cells showed an increasing trend (**Figure 5B**), although there was no significant difference compared to the proportion at BF. However, in patients whose CD8+CD39+ T cells increased at 1W, 62.5% (10/16) patients were free of recurrence 1 year after ablation, while only 20% (2/10) patients did not relapse 1 year after ablation were patients whose CD8+CD39+ T cells did not increase at 1W (P=0.051) (**Figure 5C**). Furthermore, patients with increased CD8+CD39+ T cells at 1W had better survival than those without increased CD8+CD39+ T cells at 1W post-ablation (P=0.016) (**Figure 5D**). Unfortunately, the mildly increased CD8+CD39+ T cells at 1W (0.66 ± 0.58%) decreased significantly at 4W after ablation (0.22 ± 0.47%) (P=0.0078) (**Figure 5E**). These findings suggest that, as indicated by CD8+CD39+ T cells, ablation could trigger a transient induction of anti-tumour specific T cell immunity.

Table 3ACharacteristics of patients who was detected the BF:1W matched CD8+CD39+ T cells.

**Table:** 

Characteristic (n=26)	BF	1W	P
Gender (male/female)	19/7		
Age	54.73 ± 9.35		
Pathogeny (HBV/other)	24/2		
Liver cirrhosis (no/compensated/decompensated)	8/16/2		
Differentiation (well/moderate/poor/ND)	2/4/1/19		
BCLC stage (0/A/B)	5/16/5		
WBC (10^9^/L)	5.44 ± 0.35	6.13 ± 1.06	0.111
HGB (g/L)	143.25 ± 4.41	130.89 ± 7.45	**0.033**
PLT (10^9^/L)	154.21 ± 12.93	187.44 ± 45.65	0.893
ALT (U/L)	30.17 ± 3.09	111.78 ± 24.29	**0.000**
AST (U/L)	32.38 ± 4.00	62.67 ± 11.40	**0.000**
TBiL (μmmol/L)	16.50 ± 1.99	16.70 ± 2.74	0.962
ALB (g/L)	41.13 ± 1.02	34.78 ± 1.66	**0.000**
PT (s)	11.85 ± 0.28	13.39 ± 0.71	**0.027**
PTA (%)	94.04 ± 3.34	78.89 ± 5.41	**0.025**
AFP (ng/mL)	385.57 ± 307.99	118.90 ± 107.57	0.487
PIVKA-II (mAU/mL)	1571.04 ± 803.51	49.44 ± 11.11	0.075

**Table 3B:** Characteristics of patients who was detected the BF:1W:4W matched CD8+CD39+ T cells.

**Characteristic (n=8)**	**BF**	**1W**	**4W**	**P_a_**	**P_b_**	
Gender (male/female)	6/2					
Age	52.13 ± 8.10					
Pathogeny (HBV/other)	8/0					
Liver cirrhosis (no/compensated/decompensated)	1/7/0					
Differentiation (well/moderate/poor/ND)	1/2/0/5					
BCLC stage (0/A/B)	0/6/2					
WBC (10^9^/L)	5.60 ± 1.82	6.09 ± 2.30	4.87 ± 2.30	0.207		0.293
HGB (g/L)	146.88 ± 23.12	128 ± 34.76	141.80 ± 23.64	**0.046**		0.875
PLT (10^9^/L)	151.75 ± 59.61	149.50 ± 64.05	139.40 ± 56.95	0.674		0.834
ALT (U/L)	36.13 ± 24.13	110 ± 73.79	27.40 ± 9.76	**0.004**		0.689
AST (U/L)	39.50 ± 30.98	71.50 ± 34.69	36.80 ± 23.79	0.128		1
TBiL (μmmol/L)	14.41 ± 2.49	15.75 ± 5.05	18.14 ± 9.71	0.834		0.862
ALB (g/L)	40.73 ± 4.72	32.95 ± 6.80	39.92 ± 5.58	**0.018**		0.728
PT (s)	12.04 ± 1.36	13.68 ± 2.94	12.54 ± 1.81	0.496		0.826
PTA (%)	91.63 ± 16.93	78.25 ± 21.84	86 ± 16.31	0.444		0.883
AFP (ng/mL)	958.32 ± 2599.09	252.71 ± 484.43	24.80 ± 41.23	0.245		0.172
PIVKA-II (mAU/mL)	4016.75 ± 6297.27	61.75 ± 46.82	53.80 ± 71.10	0.061		**0.005**

HBV, hepatitis B virus. WBC, White Blood Cell. HGB, hemoglobin. PLT, platelet. ALT, alanine aminotransferase. AST, aspartate aminotransferase. TBil, total bilirubin. ALB, albumin. PT, prothrombin time. PTA, prothrombin activity. AFP, alpha-fetoprotein. PIVKA-II, protein induced by vitamin K absence or antagonist-II.

Data were expressed as mean ± SD. a: The data of the 1W group was compared with the BF group, P<0.05. b: The data of the 4W group was compared with the BF group, P<0.05.

## Discussion

As a first-line treatment for HCC, the immunostimulatory effect of ablation has been examined in both patients and animal models. However, 70% of patients still inevitably relapse after ablation treatment, indicating that immune-related mechanisms deserve more detailed investigation.

This study showed that the immune responses of T cells were distinct at different time points of therapy, before and after ablation. Using of a variety of TAAs viewing as a whole to detect specific T cells, we determined that the magnitude of the T cell response, the frequency proportion of T-cell recognition, as well as the T cell response profile showed an obvious difference in the TAA-specific T cell response between patients before and after ablation, indicating that TAA-specific T cell responses were significantly affected by the ablation treatment.

We then further analyzed the data of the patients at different time points after ablation therapy. Data one week post-ablation showed that the magnitude of TAA-specific T cell responses had a decreasing trend with respect to the pre-ablation timepoint. The profile of TAA-specific T cell response changes to that dominated by SMNMS-specific T cell response. As our previous study highlighted, the immunodominance of the SMNMS-specific T cell response was a symbol of early stage immune-responsive HCC and could protect HCC from recurrence (14). Furthermore, additional analysis showed that patients with “SMNMS+ AFP-” specific T cell response one week after ablation had a longer RFS from HCC recurrence.

Interestingly, the AFP-specific T cell response was not elicited in any of the patients at one week after ablation. Indeed, the AFP-specific T cell immune response disappeared in almost half of the patients (42.86%) one week after ablation. In our previous study (14), the immune response of AFP-specific T cells was found to be associated with tumor progression, whereas the immune response of SMNMS-specific T cells had a protective effect in patients with early onset HCC. The change in the immune response profile suggested that the immune response of T cells switched toward a direction conducive to tumor control after ablation. In fact, the survival of patients with a negative AFP-specific T cell immune response was significantly better than that of patients with a positive AFP-specific T cell immune response. Furthermore, patients with a high SMNMS-specific T cell immune response achieved a longer survival than patients with a low T cell immune response.

Furthermore, to better analyze the status of T cells, we detected the phenotypic status of specific T cells before and after ablation as represented by the expression of the phenotypic markers PD1 (15) and Tim3 (16), which are commonly used to assess T cell exhaustion. The percentage of CD8+PD1+ and CD8+Tim3+ T cells decreased significantly at one week after ablation, indicating that T cell exhaustion was significantly reduced and that potentially active T cells was enhanced at this time, reflecting the improvement effect of ablation therapy on T cell immunity. Briefly, the specific antitumor ability of T cells can be revitalized by thermal ablation. Prognostic analysis revealed that the patients had already developed immunological downstaging at the time of one week.

However, this immune response was not durable. Four weeks after ablation, the proportion of patients who developed an AFP-specific T cell immune response was the highest than any other type of T cell immune response, and the proportion of patients who had lost the AFP-specific T cell immune response was very small. Moreover, the cell levels of CD8+PD1+ and CD8+Tim3+ T cells increased significantly after 4W. These results suggested that the antitumor T cell immune evoked by ablation was transient within a month period.

Furthermore, CD39, which is defined as a marker of tumor-specific CD8+ T cells in the tumor microenvironment (17), and an effective peptide-induced antitumor response has been reported to be related to activation of CD39+CD8+ T cells in PBMCs of patients with HCC (18). Therefore, changes of CD8+CD39+ T cells and be an approach to validate the immunoenhancement effect induced by ablation. Interestingly, although there was only an increasing trend of CD8+CD39+ T cells from BF to 1W, these increased CD8+CD39+ T cells at 1W improved the post-ablation prognosis. This suggested that the antitumor immune response improved significantly at 1W. However, mildly increased CD8+CD39+ T cells at 1W decreased significantly by 4W. Overall, these results confirmed that the ablation-induced antitumor immune response could not be auto-sustained.

Clinically, ablation is not sufficient to prevent tumor recurrence, suggesting that the duration and function of induced tumor-specific T cells are inadequate. Therefore, besides immune escape (19), the weak induction of long-lived T cells (5), but also the inadequate stimulatory effect of ablation itself, and what is the most important is that the patients are still exposed to *de novo* carcinogenesis on their internal microenvironments and external living environments that may contribute to tumor recurrence after ‘curative’ treatment (3). Therefore, although the immune stimulation effect of ablation therapy is beneficial (5, 20), it is not durable and cannot be maintained effectively for the extended time needed to eliminate tumor recurrence in cancer candidates. Previous animal studies have also shown that ablative therapy can only stimulate the immune response to play an antitumor protective role in the short term (8), and our results further confirm this view. Furthermore, the effect of ablation on T cell immunity varies greatly between individuals, and the immunological characteristics of populations that can stimulate effective and sustained antitumor immunity deserve further study.

Together with these results, the present study suggests that HCC ablation induced transient functional activation of specific T cells, and the changes in TAA-specific T cells induced by thermal ablation should be further enhanced using additional immunological treatment approaches. In recent studies of immunology-related measures, immunomodulatory antibodies such as anti-PD1 (8, 21–23) have been considered to reactivate T cell function. This study also suggests that this approach may also be a promising option.

In conclusion, the results of this study show that ablation therapy of HCC can improve TAA-specific T cell responses and that the induced change is associated with a short-term improvement in RFS. To sustain ablation-induced TAA-specific T cell responses and improve the immunological effects on HCC, additional combined treatment with immune checkpoint inhibitors may be useful.

## Data availability statement

The original contributions presented in the study are included in the article/supplementary material. Further inquiries can be directed to the corresponding authors.

## Ethics statement

The studies involving human participants were reviewed and approved by the Institutional Review Board of Beijing YouAn Hospital, approval number [LL-2019-004-K]. The patients/participants provided their written informed consent to participate in this study.

## Author contributions

CZ: Conceptualization, methodology, investigation, formal analysis, writing-original draft preparation. YZ: Conceptualization. GL: Methodology. KL: Investigation, funding acquisition. LQ: Methodology. YZ: Supervision, revision. JS: Methodology. QW: Software. LM: Investigation. PZ: Investigation. YS: Investigation. DG: Investigation. CY: Investigation. TD: Conceptualization, methodology. YHZ: Conceptualization, supervision, project administration, funding acquisition. All authors contributed to the article and approved the submitted version.
